# Phenylketonuria patients’ and their parents’ knowledge and attitudes to the daily diet - multi-centre study

**DOI:** 10.1186/s12986-017-0207-1

**Published:** 2017-08-17

**Authors:** Ewa Witalis, Bozena Mikoluc, Radoslaw Motkowski, Jolanta Sawicka-Powierza, Agnieszka Chrobot, Bozena Didycz, Agata Lange, Renata Mozrzymas, Andrzej Milanowski, Maria Nowacka, Mariola Piotrowska-Depta, Hanna Romanowska, Ewa Starostecka, Jolanta Wierzba, Magdalena Skorniewska, Barbara Iwona Wojcicka-Bartlomiejczyk, Maria Gizewska, Halina Car, Polish Society of Phenylketonuria, Agata Lange, Agnieszka Chrobot, Andrzej Milanowski, Barbara Iwona Wojcicka-Bartlomiejczyk, Bozena Didycz, Ewa Starostecka, Hanna Romanowska, Jolanta Wierzba, Magdalena Skorniewska, Maria Nowacka, Mariola Piotrowska-Depta, Renata Mozrzymas

**Affiliations:** 1 Medical and Rehabilitation Centre in Gdansk, Gdansk, Poland; 2 Department of Pediatrics, Rheumatology, Immunology and Metabolic Bone Diseases, Medical University of Bialystok, ul. Waszyngtona 17, 15-274 Bialystok, Poland; 3 Department of Family Medicine, Medical University of Bialystok, Bialystok, Poland; 4 Brudzinski Children’s Hospital in Bydgoszcz, Bydgoszcz, Poland; 5 Polish-American Institute of Pediatrics, Cracow, Poland; 6 Polish Mother’s Memorial Hospital, Lodz, Poland; 7 Department of Pediatrics, Regional Specialis Hospital, Research and Development Centre, Wrocław, Poland; 8 National Institute of Mother and Child, Warsaw, Poland; 9 Department of Pediatrics, Endocrinology, Diabetology, Metabolic Diseases and Cardiology, Pomeranian Medical University, Szczecin, Poland; 10 Department of Pediatrics, Hematology and Oncology Medical University of Gdansk, Gdansk, Poland; 11 Medical Department Nutricia Poland, Warsaw, Poland; 12 Department of Experimental Pharmacology, Medical University of Bialystok, Bialystok, Poland

**Keywords:** Phenylketonuria, Diet compliance, Control diet, Education, Phenylalanine and foods

## Abstract

**Background:**

The aim of the study was to assess both patients’ and their parents’ knowledge of phenylketonuria (PKU) treatment and compliance with PKU diet.

**Methods:**

The study included 173 PKU patients aged 10–19 and 110 parents of PKU children who were enrolled in the study on the basis of questionnaire data. The study also included 45 patients aged ≥20.

**Results:**

Our study demonstrated that only 45% (*n* = 74) of PKU patients knew daily Phe intake recommendations, 27% of patients (*n* = 41) knew the Phe content in a minimum of three out of four researched food products. Patients’ knowledge concerning Phe intake (*p* = 0.0181) and the knowledge of selected food products (*p* = 0.041819) improved with age. We did not establish such a correlation in the group of PKU children’s parents.

Approximately 31% of patients and 22% of parents reported helplessness, which increased with the child’s age, associated with the necessity to adhere to the diet; 30% of patients reported feeling ashamed of the fact that they could not eat all food products. Regardless of age, children were more likely than parents to report helplessness (*p* = 0.032005).

Among patients, 41.40% declared that they would wish to select products unassisted but their parents did not permit them to do so. The question of whether parents teach children self-reliance in meal preparation was answered affirmatively by 98% of parents and only 81% of children (*p* = 0.0001).

**Conclusion:**

Our data demonstrated that parents’ and children’s knowledge concerning treatment recommendations and food products does not have a direct impact on attitude to the PKU diet. Limiting children’s independence in meal selection, growing helplessness in the face of dietary adherence and shame resulting from the necessity to follow a different diet observed in PKU families are responsible for shaping and perpetuating a consistently negative attitude to the diet. The care of PKU paediatric patients requires consistent, long-term family and individual therapy which may counteract the effects of learned helplessness. In regard to the educational effort, a good parent-child relationship as well as the teaching of behaviours motivating patients to comply with the diet are of great importance.

**Electronic supplementary material:**

The online version of this article (doi:10.1186/s12986-017-0207-1) contains supplementary material, which is available to authorized users.

## Background

Phenylketonuria (PKU) is the first inborn error of metabolism in which dietary therapy has been used successfully [1]. In countries which have implemented neonatal screening programmes for PKU, severe intellectual disability in PKU patients resulting from lack of treatment has become a thing of the past [2–4]. Dietary therapy initiated in the neonatal period enables patients to reach their full intellectual and social potential. However, despite the high efficacy of dietary therapy and the continuous improvement of PKU formula and supplements, adherence to the strict rules of nutritional regimen remains problematic for parents, patients and professionals.

Observing dietary rules requires relevant knowledge and adherence to the recommendations, initially by parents and, subsequently, by adolescent and adult patients. The attitude adopted by carers and patients frequently determines whether and how therapists’ recommendations are followed. Literature contains reports regarding socioeconomic and psychosocial factors which positively affect patients’ compliance with dietary recommendations and also those which impact on PKU therapy discontinuation [5–9]. There are few studies, however, regarding PKU patients’ and their parents’ knowledge of PKU therapy and its impact on dietary adherence. Nevertheless, the significance of factors occurring within the family and their influence on treatment compliance has frequently been emphasised. This justifies the need to examine the level of knowledge among PKU patients and their parents and their perception of their own abilities and limitations.

Therefore, the study attempted to answer the question of whether the awareness of dietary recommendations and knowledge of the nutritional content of selected food products impacts on attitude towards dietary compliance. Attitude towards the PKU diet was defined as a set of beliefs about the possibility of selecting food products unassisted, a feeling of shame associated with dietary restrictions, and a feeling of helplessness arising from: the necessity to continuously control the diet and the realisation of one’s inability to change the situation. The study investigated the knowledge of the daily Phe and protein requirements and the Phe content in selected food products among PKU patients aged 10 and over, and their parents.

### Materials

The study included 173 individuals with classic PKU (87 females and 86 males) aged 10–19 and 110 parents of PKU children in this age group. Additionally, the study included 45 patients aged = > 20. Study participants were inhabitants of different parts of Poland managed in nine specialist metabolic clinics in the country.

The following inclusion criteria were applied: PKU diagnosed in early infancy in a newborn screening test (at follow-up tests blood Phe concentration > 1200 μmol/L), exclusion of atypical forms of PKU (loading tests with phenylalanine and tetrahydrobiopterin, urinary pterin profiles), a low phenylalanine diet from early infancy, intellectual development within age norm. According to the International Standard Classification of Education (ISCED 1–7 level) the patients were in primary, secondary and higher education. Individuals suffering from mild PKU (at follow-up tests blood Phe concentration 600–1200 μmol/L), and other chronic diseases were excluded from the study.

The patients and their parents were routinely instructed on the principles of dietary therapy including the individual intake of phenylalanine (mg/day) and protein (g/day), and the quantity (g/day) and frequency of PKU formula intake (≥3×/day). Nutrition education also involved acquiring knowledge of menu planning and Phe, protein and calorie content in food products. Characteristics of the PKU patients and parents are presented in Table 1 and Table 2.

**Table 1:** Characteristics of PKU patients (*n* = 218)

Age at study (years)	*N*	*%*	Male		Female	
			*N*	*%*	*N*	*%*
10–13	70	40.46	38	54.29	32	45.71
14–16	70	40.46	30	42.86	39	55.71
17–19	33	19.08	18	54.55	16	48.48
	173		86	49.71	87	50.29
> = 20	45		24	53.33	21	46.67
Total	218		110		108

**Table 2:** Characteristics of the cohort of PKU parents (*n* = 110)

	Number	Percent
Age of child at study (years)		
10–13	50	45.45
14–16	42	38.18
17–19	18	16.36
Sex of child		
Female	61	55.45
Male	49	44.55
Place of residence		
Rural	32	29.09
Urban	78	70.91
Economic conditions of family		
Poor	1	0.91
Average	22	20.00
Good	75	68.18
Very Good	8	7.27
No data	4	3.64
Educational level of mathers^a^		
Low	4	3.64
Vocational	17	15.45
Middle	60	54.55
High	29	26.36
No data	4	3.64
Educational level of fathers^a^		
Low	4	3.64
Vocational	38	34.55
Middle	42	38.18
High	30	27.27
No data	6	5.45

^a^ Low: primary education. Vocational: technical training. Middle: middle vocational education, secondary education. High: higher vocational education, university

The study was approved by the Bioethical Committee at the Medical University, Bialystok, Poland (Nr R-I-002/496/2016). Each participant signed informed consent to participate in the study.

## Methods

The study was conducted during regional and national meetings of PKU families with therapists. Participation in the survey was voluntary. Members of PKU families not participating in the meetings were not approached and asked to take part in the study.

The PKU patients and their families were enrolled in the study on the basis of questionnaire results. The questionnaire designed by the research team in cooperation with clinical psychologists assisting PKU patients and their families, and experts from the Polish Society of Phenylketonuria. The questionnaire completed by the study participants has been attached (Additional file 1).

The questionnaire contained nine questions on the patients’ and their parents’ attitudes to the disease. The questions concerned the knowledge of daily Phe and protein intake and the Phe content in four selected food products (apple, tomato, potato, a slice of low protein bread). The participants were considered knowledgeable about the Phe content in food products if they provided the correct response to questions regarding at least three food products. The study also evaluated the subjects’ capability and possibility of selecting appropriate food products unassisted, a sense of helplessness regarding the necessity to continuously control the diet and a feeling of shame caused by the need to follow a different diet.

Questions for parents and children were similar in format. A Likert-type response scale was used in the survey. Children below the age of 15 completed the questionnaire either unassisted or with the help of a qualified researcher. The family’s socioeconomic status was only assessed by the parents.

### Statistical analysis

All calculations were performed using the Microsoft Excel spreadsheet and STATISTICA, StatSoft, Inc., version 8.0 statistical package (data analysis software system). Statistical evaluation of quantitative data utilised the classical measures of location such as arithmetic means and medians, and measures of variation such as standard deviation and range. Normality distribution of the variables and variance equality of studied features in groups was established with the use of Shapiro–Wilk test and variance equality test. In order to compare groups in pairs for quantitative data, t test or Mann–Whitney test were used with respect to the type of distribution of the variables tested. In the case of multiple group comparison, the Kruskal–Wallis test was used as the nonparametric equivalent of one-way analysis of variance (ANOVA). In all these calculations, the statistical significance level was set at *p* < 0.05.

## Results

The studied group of patients followed the rules of classic PKU therapy applied in Poland [10]. Phe intake ranged from 200 to 900 mg/day and was set on the basis of individual tolerance and the blood phenylalanine concentration level recommended for a particular age [4, 11, 12]. The study demonstrated that the recommended protein intake based on the patient’s weight and age ranged from 40 to 80 g/day. The phenylalanine-free mixture of amino acids (PKU formula in the form of powder, liquid or tablets) provided 80–90% of the daily protein requirement. The participants reported the lowest and highest blood phenylalanine concentration levels in the last year of therapy. Study results analysis did not reveal correlations between blood phenylalanine levels and the researched parameters.

**Fig. 1:**
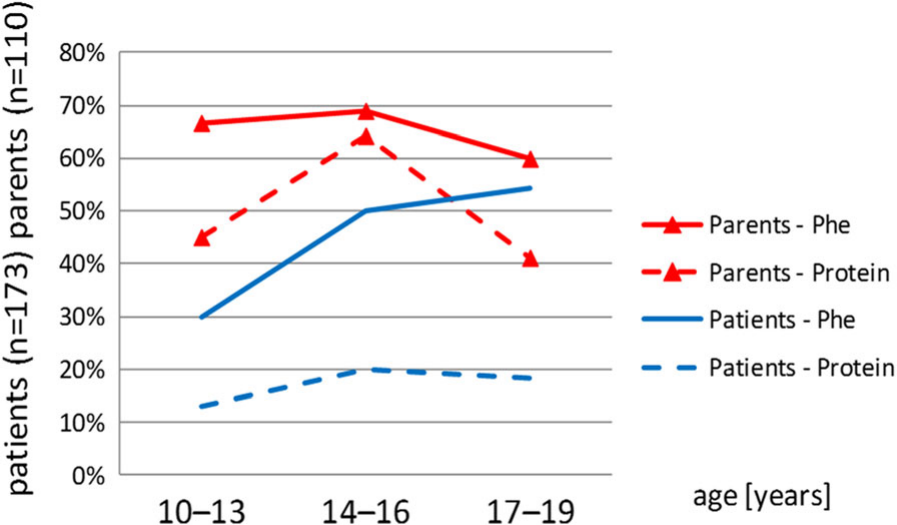
Patients’ and parents’ knowledge of the daily requirement of phenylalanine (Phe) and protein

The present study involving 173 PKU patients aged 10–19 years demonstrated that only 44.83% (*n* = 74) of patients knew the recommendations concerning the daily Phe requirement and 29 patients (17.03%) knew the daily protein intake. Analysis of the obtained results revealed that the knowledge of the daily Phe intake increased with the child’s age (*p* = 0.0181), whereas no statistically significant differences in the level of knowledge regarding the recommended protein intake between particular age groups were found (*p* = 0.5121) /Fig. 1/. Research results concerning parents revealed that in the group of 110 parents, 65.23% (*n* = 72) stated that they knew the daily Phe requirement, and 50.2% (*n* = 57) also knew the recommended protein intake /Fig. 1/. The study results demonstrated that the parents of children under the age of 16 years possessed a greater awareness of protein intake than the children (age group 10–13 years *p* = 0.0001) (age group 14–16 years *p* = 0.0001) and that the parents’ knowledge of Phe intake was also greater (age group 10–13 years *p* = 0.0001) (age group 14–16 years *p* = 0.0486). Statistical analysis did not show differences in the knowledge concerning the daily Phe (*p* = 0.8158) and protein (*p* = 0.1160) intake in the studied groups of parents /Fig. 1/.

As far as the family’s socioeconomic status is concerned, over 70% of the 110 parents of PKU patients described their family’s economic conditions as very good or good, 20% as average and 1% as poor. More than 70% of parents had vocational or secondary education, 25% had higher education and 4% had primary school education /Table 2/.

**Fig. 2:**
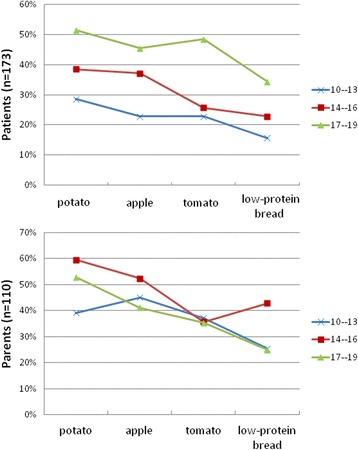
Patients’ and parents’ knowledge about content of Phe in products (mg/100 g)

In response to the question regarding the Phe content in 100 g of four most commonly used food products (apple, tomato, potato, a slice of low-protein bread), only 27.2% of patients (*n* = 41) knew the Phe content in three or four products /Fig. 2/.

A comparable level of knowledge in this area was demonstrated by the patients’ parents since 24.2% knew the Phe content in a minimum of three out of the four products researched. Patients aged 17–19 years possessed a higher level of knowledge concerning the Phe content in four selected products than patients aged 14–16 years (*p* = 0.041819). Correct answers were most frequently provided by patients aged 17–19 years (44.12%) and the parents of children aged 14–16 (30.95%). In all the age groups, both patients and parents most frequently provided the correct answer to the question regarding the Phe content in a potato (39.55% of patients; 50.56% parents) and least frequently in a slice of low protein bread (24.32% patients; 31.12% parents) /Fig. 2/.

**Fig. 3:**
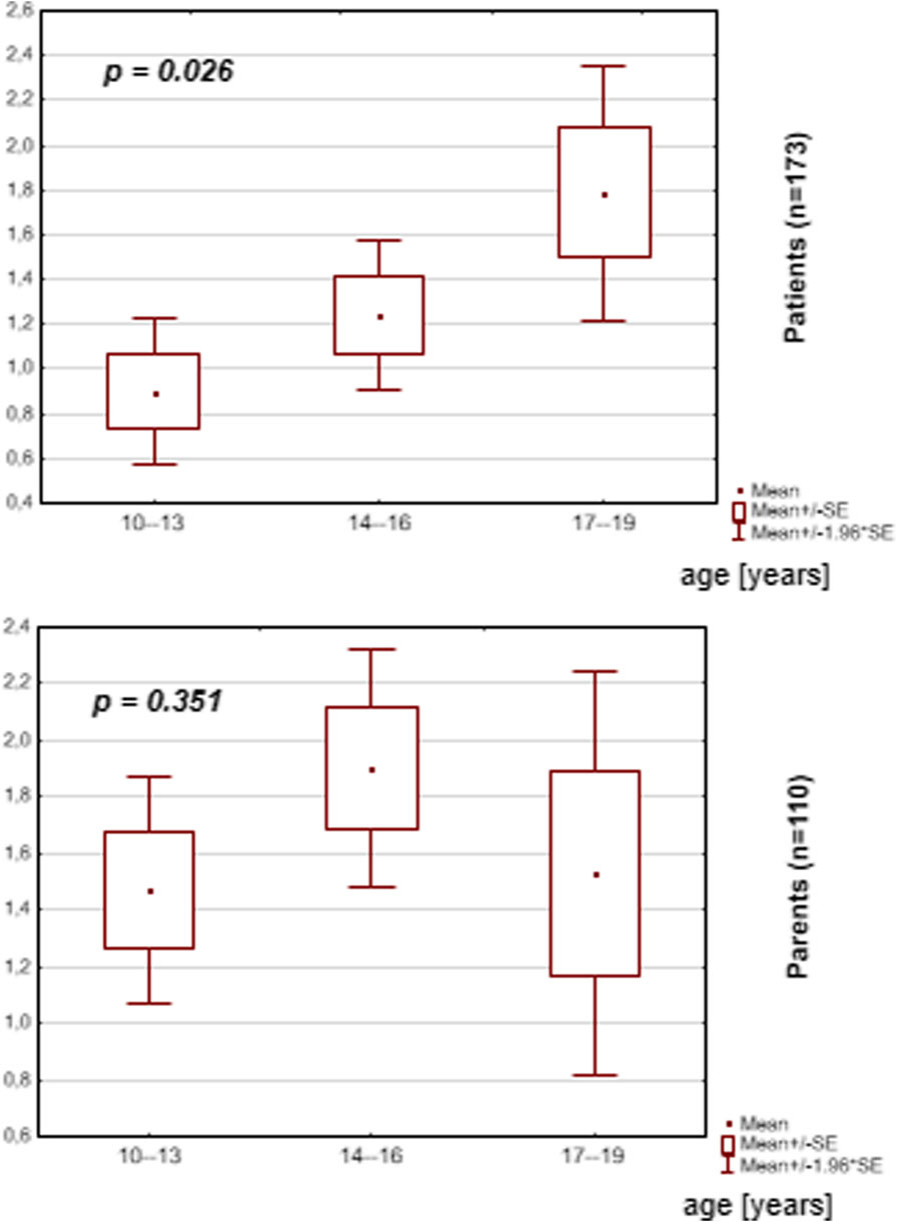
Patients’ and parents’ knowledge of Phe content (mg/100 mg) in four selected products

It should be emphasised that patients above the age of 14 years old possess a higher level of knowledge concerning the daily Phe intake (*p* = 0.0181) and have a greater awareness of the Phe content in four selected food products (*p* = 0.0258) in comparison with patients aged 10–13 years /Fig. 3/.

However, no differences, irrespective the child’s age, were found between the studied groups of parents regarding knowledge of the daily intake of Phe (*p* = 0.8158) and protein (*p* = 0.1160), and the Phe content in selected food products (*p* = 0.3509) /Fig. 3/.

As far as the question relating to the frequency of formula intake is concerned, 73.8% of parents and 75.41% of children believed the intake to be three /≥3/ portions per day. The correct answer was most frequently provided by patients aged 14–16 years (82.61%) and the parents of children aged 10–13 years (84%). Only 58% of parents of children aged 17–19 years declared formula intake to be ≥3× per day.

Our study also demonstrated that a sense of helplessness regarding the necessity to control the diet was reported by 22.13% of parents and 30.75% of patients.

Helplessness was more frequently reported by children than by parents irrespective of the studied patients’ age (*p* = 0.032005).

A feeling of helplessness was reported by 21.4% of children aged 10–13 years and 39.4% of children aged 17–19 years. In the group of patients, no correlation was established between the knowledge of dietary recommendations and the feelings of helplessness.

**Fig. 4:**
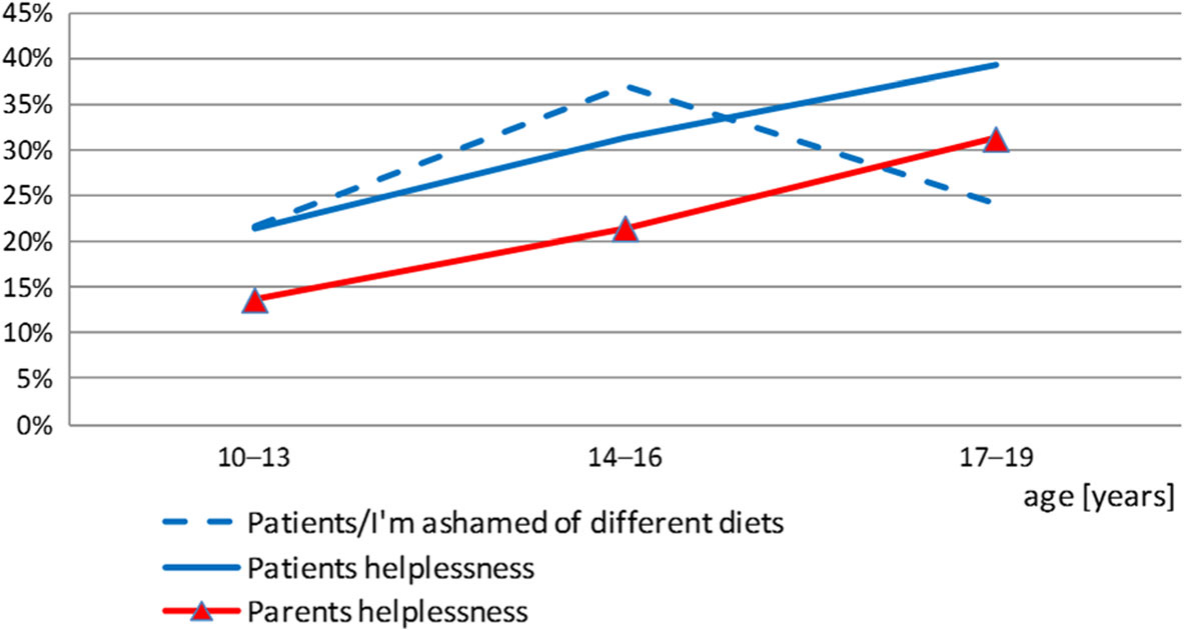
Patients’ and parents’ helplessness in face of the need to control diet and patients shame due to different meals

Data analysis revealed that the parents’ and children’s sense of helplessness concerning the necessity to follow a special diet increased with the child’s age /Fig. 4 /

In the studied groups of parents no correlation was established between the knowledge of dietary recommendations and the Phe content in four selected food products, and the sense of helplessness concerning the need of dietary control.

The question of whether parents teach children self-reliance in meal preparation was answered affirmatively by 98.18% of parents and only 80.92% of children (*p* = 0.0001). It is noteworthy that the affirmative answer was provided by 100% of parents of children aged 17–19 years and only 78.8% of patients (*p* = 0.0302).

Our study demonstrated that 41.4% of patients expressed a wish to select food products unassisted more frequently but their parents did not allow them to do so. It is interesting that as many as 48.5% of children aged 17–19 years, and 41.4 and 34.3% of children aged 10–13 years and 14–16 years respectively expressed this opinion. The question of whether children were capable of choosing foods permitted on their diet was answered affirmatively by 97.63% of parents and 90.78% of children. However, only 44.83% of children knew the daily dietary Phe intake and only 32.84% of children were aware of the Phe content in four selected food products. It should be emphasised that although 100% of the surveyed parents claimed that their children were capable of selecting food products unassisted, it was only 30% of children aged 10–13 years and 54.5% of patients aged 17–19 years who knew the daily dietary Phe intake and only 22.5 and 44.96% respectively knew the Phe content in staple foods of their diet.

The study also explored the aspect of shame associated with dietary restrictions. The study demonstrated that 29.51% of patients and 34.5% of parents reported that their children were ashamed of not being allowed to eat all meals. The view was expressed most frequently by adolescents aged 14–16 years (37.1%) and parents of children aged 17–19 years (41.2%) /Fig. 4/.

The study revealed a lack of correlation between the feeling of shame associated with the necessity to follow a different diet, and the knowledge of dietary recommendations and the Phe content in food products. A comparative analysis of the groups of 45 PKU patients aged 20 years and above and 173 PKU patients aged 10–19 years is noteworthy. The analysis revealed that adult patients had a greater knowledge of Phe (*p* = 0.0390) and protein (*p* = 0.0055) intake recommendations, and a greater awareness of the Phe content in two out of four selected food products (tomato *p* = 0.0467) (apple *p* = 0.0475). Furthermore, only 33.33% of adult patients knew the Phe content in a minimum of three selected food products and 84.44% of adult patients named formula intake recommendations as ≥3×/per day /data not shown/. The study demonstrated that among adult patients 38.6% reported a sense of helplessness and 42.2% reported that parents did not permit them to choose foods unassisted. It is also noteworthy that adult patients were twice as likely as adolescents aged 17–19 years to report feeling ashamed about their different diet (48.9% versus 24.2%).

## Discussion

### Knowledge of PKU diet

Our research demonstrated that in the group of 173 of PKU patients aged 10–19 years and 110 parents, it is the parents who possessed a greater knowledge of recommendations concerning the daily Phe and protein dietary intake and a greater knowledge of the Phe content in four selected food products in comparison with the children. It was established that only in the studied children there was a statistically significant, age-related increase in the knowledge regarding the Phe intake and content in food products. The study demonstrated that only approximately 27% of children and 24.2% of parents knew the Phe content in three out of four selected food products. We established that in the group of parents the frequency of correct responses decreased with their child’s age. Our study also revealed that both the parents and the children knew the recommended daily formula intake levels (three or more portions per day).The obtained results are in agreement with the data acquired in studies on mothers of PKU children which demonstrated that poor parental dietary knowledge may have an impact on long-term metabolic control in children [13]. The available literature indicate that maternal knowledge of the exchange system (15 mg Phe) is correlated with blood Phe concentration in children even up to the age of 15 years. There was a negative correlation between maternal knowledge about exchange and median blood Phe concentration in the child [14].

Three international surveys confirmed a discrepancy between clinicians and patient views regarding the perceived effectiveness of the nutrition education tools. A consistent decline in ‘parents as role models’ as an educational tool was observed starting at age 10 years. Patients responded they feel their families are the most effective form of education [15].

The results of our research indicate the need to develop interactive educational programmes regarding the Phe content in food products *because* only 33 to 40% of patients knew the Phe content in the most commonly products like apple, tomato or potato. Literature reports indicate that educational interventions yield a long-term improvement in dietary knowledge but only a short-term improvement in metabolic control [16].

### Feelings of helplessness and shame regarding PKU diet

This acknowledges the need to incorporate regular, long-term cognitive-behavioural therapy into the care of PKU paediatric patients, particularly since negative attitudes and emotions associated with the necessity for dietary adherence may exist in PKU families [17].

An attempt to answer the question of whether the level of knowledge regarding PKU therapy impacts on the sense of helplessness and shame connected with the necessity to control the diet, which was observed in PKU families, constituted an important aspect of our study.

Our research demonstrated that approximately 22% of parents and 30% of children reported a sense of helplessness associated with the need to control the diet. Data analysis demonstrated that the knowledge of dietary recommendations did not influence the sense of helplessness associated with the need to control the diet. Feelings of helplessness were experienced by both parents and children despite a steady growth in the level of dietary awareness among patients. Therefore, further studies are needed to identify factors causing the sense of helplessness in PKU families [18].

Research confirms the need for professional actions focusing on imparting the practical knowledge of PKU nutrition in the family [19]. There is a need for forming support groups and family therapy programmes in order to overcome negative attitudes towards the PKU diet [20, 21]. This concerns not only the sense of helplessness but also the feeling of shame evoked by the necessity to follow a different diet, which was reported by approximately 30% of patients under the age of 19 years and was not related to their knowledge. We observed a tendency for the feelings of shame to increase, particularly among patients above the age of 20 years. Studies by Levy et al. have demonstrated that in PKU adolescents: rationale and psychosocial factors are most highly related to metabolic control and for the social support for the diet and positive perceptions of treatment [22].

Other researchers have also confirmed that an improvement in the knowledge regarding therapy is not closely correlated with improved dietary compliance [16]. This claim is corroborated in the studies by Durham-Shearer et al. which demonstrate that most adolescents and adults with phenylketonuria were aware of dietary recommendations, although this did not always result in compliance. The improvement in knowledge was not accompanied by a significant improvement in measures of compliance [23]. Diet-related problems and negative emotions observed in PKU families may suggest a failure to incorporate therapy into daily life [24]. It has been demonstrated that in families who perceive the diet as a challenge children have elevated serum Phe concentrations. Additionally, short-term effects of the education intervention resulted in improved metabolic control associated with improved attitudes, increased knowledge of diet and disease, increased perceived support, and decreased barriers to dietary compliance in a camp setting [16].

### Adolescent and adult PKU patients

Our research indicates that adolescent patients’ conviction about the permanence of problems arising from the necessity to adhere to the PKU diet does not conduce to dietary compliance. Restrictions experienced by the patient may result in cognitive and motivational disturbances [25, 26]. Our results prove that the parents of children aged 10–19 years significantly limit the patients’ opportunity to select food products. The study demonstrated that among adolescent patients, who declared the ability to choose PKU diet appropriate foods, approximately 40% reported that they would like to select food products unassisted but their parent did not permit them to do so. It is particularly noticeable in the group of 17 to19-year-old patients, approximately 50% of whom reported these restrictions. Despite an improvement in the knowledge of therapy, patients do not choose meals unassisted in their parents’ presence. Such attitudes perpetuate models of behaviour which heighten the sense of helplessness and shame, and have a negative impact on children’s perception of own capabilities. This results in adolescent patients’ inability to effectively apply their knowledge in practice when faced with situations of unassisted meal choices outside of the home. This is important since in the process of growing up adolescents contend with external psycho-social conditions which further reduce the possibility of following the diet outside of the home [8]. Limiting opportunities to select meals at home and the embarrassment felt as a result of following a different diet have a detrimental effect on shaping adolescents’ attitudes towards the PKU therapy, thus fostering learned helplessness. Our research results had been corroborated by other authors who have demonstrated that suffering from a chronic metabolic disease with the necessity of parental control of behaviour and diet often results in overprotection in childhood with a delay in achieving autonomy [27, 28]*.* This indicates the need for shaping and promoting the right attitudes towards dietary recommendations in the family. Our study demonstrated that all the surveyed parents declared that they taught their children independence, 80% of whom confirmed the claim.. Parents should receive practical advice on how to act in order to reduce feelings of helplessness and shame and foster their children’s independence. The results analysis, however, revealed that in comparison with their parents children always underestimate their ability to select meals Our research results, similarly to literature data, confirm the need for undertaking targeted educational interventions aimed at eliminating patients’ erroneous belief [29]. Behavioural interventions involving the shaping of required behaviours have proven to be efficacious [30]. Similar conclusions can be drawn from studies which focused on the impact of psychosocial and emotional factors on dietary compliance [31]. The results analysis concerning patients above the age of 19 years constitutes an important finding of our study. The surveyed adult PKU patients reported significantly heightened feelings of shame associated with a different diet, and the feelings of helplessness associated with the necessity to follow a dietary regimen were reported with the same frequency as by adolescents. This finding confirms the profound impact of models of behaviour within the family on adult patients’ attitudes towards the diet and may be one of the reasons for non-compliance [32]. The question of how to provide PKU families with an integrated, unified and comprehensive care system which will assist in achieving dietary compliance among adolescent and young adult patients remains unanswered. Forming support groups for PKU families, offering behavioural therapy and providing opportunities for benefitting from other methods of motivation, e.g. coaching in order to comply with dietary recommendations and achieve personal goals remains a challenge.

## Conclusion

The study, conducted on a large group of PKU patients and their parents, demonstrated that it is the parents who possess a greater knowledge of recommendations concerning the dietary Phe and protein intake and a greater awareness of the Phe content in four selected food products in comparison with the children. Our research indicates that the knowledge of treatment recommendations and food product content does not influence dietary compliance. The feelings of helplessness reported by PKU families and those of shame experienced by patients due to dietary restrictions are responsible for shaping and instilling a consistently negative attitude to the PKU diet. Professionals involved in PKU therapy ought to recognise the feelings of helplessness and shame reported by patients and make appropriate interventions. The care of PKU paediatric patients requires consistent, long-term family and individual cognitive bahavioural therapy, the formation of support groups and, additionally, interactive educational programmes. Teaching behaviours promoting patient independence in following dietary recommendations constitutes an important educational aspect in PKU families. Studies indicate that the good parent-child relationship may counteract the effects of learned helplessness and may reduce the feelings of shame associated with adherence to a different diet. Therefore, further research is needed to identify factors causing the sense of helplessness in PKU families.

## Additional file

Additional file 1: Questionnaire for PKU patients. (PDF 105 kb)

## Abbreviations

Phe: Phenylalanine
PKU: Phenylketonuria
