# Supplementary material for: Phenylketonuria patients’ and their parents’ knowledge and attitudes to the daily diet - multi-centre study
Source: Nutr Metab (Lond). 2017 Aug 17;14:57. doi: 10.1186/s12986-017-0207-1 (PMC5559804; doi:10.1186/s12986-017-0207-1)
Supplement: Additional file 1: — Questionnaire for PKU patients. (PDF 105 kb) [file 12986_2017_207_MOESM1_ESM.pdf]

Questionnaire for PKU patients

Name and surname:		
Date of birth:		
Place of residence:	urban	rural

Please state to what degree the following statements **relate to you** at this time

Answer by placing X in the relevant box

1.	I can choose food products which I am allowed to eat unassisted.	Strongly disagree	Somewhat disagree	Neither agree nor disagree	Somewhat agree	Strongly agree
2.	Parents teach me self-reliance in meal preparation.	Strongly disagree	Somewhat disagree	Neither agree nor disagree	Somewhat agree	Strongly agree
3.	I would prefer to decide more often what I can eat but my parents do not allow me to.	Strongly disagree	Somewhat disagree	Neither agree nor disagree	Somewhat agree	Strongly agree
4.	I feel helpless because of the constant need to follow the diet.	Strongly disagree	Somewhat disagree	Neither agree nor disagree	Somewhat agree	Strongly agree
5.	I am often ashamed of not being able to eat all foods.	Strongly disagree	Somewhat disagree	Neither agree nor disagree	Somewhat agree	Strongly agree

6. State your daily requirement for:

Phenylalanine (mg)		Protein (g)	

7. How many milligrams of phenylalanine are in 100 grams of:

apple		potato	
tomato		a slice of low protein bread	

8. How many times per day do you take formula:

1-2 times per day		3 and more times per day	

9. State your blood phenylalanine levels within the last twelve months:

lowest		highest	

Thank you for completing the questionnaire.
